# New method for estimating the post-mortem interval using the chemical composition of different generations of empty puparia: Indoor cases

**DOI:** 10.1371/journal.pone.0209776

**Published:** 2018-12-20

**Authors:** Michele C. Paula, Kamylla B. Michelutti, Aylson D. M. M. Eulalio, Raul C. Piva, Claudia A. L. Cardoso, William F. Antonialli-Junior

**Affiliations:** 1 Laboratório de Ecologia Comportamental, Centro de Estudos em Recursos Naturais, Universidade Estadual de Mato Grosso do Sul, Dourados, Mato Grosso do Sul, Brazil; 2 Programa de Pós-graduação em Entomologia e Conservação da Biodiversidade, Universidade Federal da Grande Dourados, Dourados, MS, Brazil; 3 Programa de Pós-graduação em Recursos Naturais, Universidade Estadual de Mato Grosso do Sul, Dourados, Mato Grosso do Sul, Brazil; 4 Programa de Pós-graduação em Química, Universidade Federal da Grande Dourados, Dourados, Mato Grosso do Sul, Brazil; Purdue University, UNITED STATES

## Abstract

Most flies of forensic importance are in two superfamilies, the Muscoidea and the Oestroidea, with similar life stages including the puparium. Upon completion of metamorphosis the adult fly emerges from the puparium, leaving behind an exuvia that is of potential significance in forensic investigation. The empty puparium is a durable piece of entomological evidence lasting several years. Through the study of chemical compounds, specifically the hydrocarbons of these puparia, it is possible to identify the species, in addition to how long they have been exposed to weathering and for this reason, these parameters can assist forensic entomologists in estimating long-term postmortem interval (minPMI). In corpses that take a relatively longer time to decompose, insects may use the same corpses for several oviposition cycles. Therefore, the aim of this study was to develop a new method to determine the PMI based on chemical compounds of the puparia from different oviposition cycles of the fly *Chrysomya megacephala*. The chemical composition of 50 puparia from different cycles of oviposition were evaluated by Gas Chromatography–Mass Spectrometry (GC-MS). In total, 60 compounds were identified ranging from C_18_ to C_34_, 38 of those were common to all generations. Our results demonstrate that chemical profiles can be used to differentiate puparia collected from successive cycles, and therefore valuable in the estimation of minPMI.

## Introduction

Blow flies (Diptera: Calliphoridae) are necrophagous insects, that use protein resources for ovary maturation, oviposition and development of their offspring [1], and are attracted primarily by the odor released in the first stages of corpse decomposition [2,3]. They are generally present in the first wave of faunistic succession, occurring in all types of decomposing remains, including human [2,4,5]. Therefore, they are considered the most important insect group for forensic entomology, for their widespread distribution, predictability, relative abundance and reliable forensic databases for reference [2,6,7].

Soon after death, thanatologic methods are a reliable tool to determine time since death, however, when decomposition has progressed to advanced decay or dried remains, entomological data may be more useful in predicting a Post Mortem Interval (PMI) [7,8]. To perform a more accurate PMI estimation, forensic entomologists use data from the insects that presumably oviposited on the body first [9].

Sometimes when the body is in a state of advanced decomposition, it is necessary to assess puparia, especially of flies, in the vicinity of the body [10–12]. Any whole live pupa would be collected and held for emergence into adult flies to confirm the identification of the collected larvae and direct the investigator to the applicable databases. Often the eclosed puparia at the scene are ignored because the morphological identification of puparia is difficult due to the lack of important features for traditional taxonomy and damages present in puparia as a result of adult emergence hindering their use [10,11]. However, when the corpse decomposition is extremely advanced the only non-corpse constituents found nearby are fly puparia [12]. Thus, the puparia may provide reliable data about the post-mortem interval because they persist in decomposed remains, even after years [10,13,14].

A complementary tool that has been used to expedite species identification is the analysis of cuticular chemical compounds. These compounds are a constituent part of the lipid layer of insects, used primarily to prevent desiccation [15], but also for chemical communication [16]. They are a mixture of linear alkanes, branched alkanes and alkenes [17]. The compounds have been shown to be affected by genetic [18,19] and environmental factors [18,20–23].

Cuticular hydrocarbons in the field of forensic entomology can be used as a complementary taxonomic tool for identification of species [10,24,25], populations [20,26–28], age, and developmental stage [10,28,29].

However, when the corpse decomposition is extremely advanced the only non-corpse constituents found nearby are fly puparia [12]. Thus, the puparia may provide reliable data about the postmortem interval because they persist in decomposed remains, even after years [13]. The chemical compounds of the cuticle of the puparia can also be used to identify the species [10] and thus help to estimate the PMI [30,31], since their chemical degradation is slow [11].

In a situations when a body is inside a residence, with restricted access to necrophagous insects, it takes longer to decompose affecting the speed of colonization and access to corpses [32], resulting in a much lower diversity as compared to substrates exposed outdoors [33,34]. Therefore, the species that gains access will oviposit and the emergent offspring from the first wave may use the corpse as a resource for ovary maturation and development of their offspring. Additionally, in temperate areas where corpses take longer to decompose at lower temperatures [35,36], the insects may use the same corpse in successional oviposition cycles [36]. In situations where the corpse remains suitable for colonization [37], there may be multiple generations of puparia associated with the corpse. Indeed, in indoor studies with human corpses, successive generations of two species of blow flies *Lucilia sericata* Meigen 1826 and *Protophormia terraenovae* Robineau-Desvoidy 1830 were found [32].

In indoor cases, in which insects can use the same corpse in successive cycles of oviposition, it is possible to find samples of puparia from different generations around the corpse. However, using only the latest samples of insects that laid eggs on the corpse can lead to imprecise determination of PMI. Therefore, the aim of this study was to develop a new method for PMI estimation, based on chemical compounds from puparia of different generations of the blow fly of forensic importance *Chrysomya megacephala*.

## Materials and methods

### Samples collection

To evaluate the variation of the chemicals in the puparia from three different generations of *C*. *megacephala* oviposition, wild adults were collected by trapping in the municipality of Dourados-MS, Brazil, on the Campus of Universidade Estadual de Mato Grosso do Sul (latitude 22° 11’ South, longitude 54° 55’ West). Traps were constructed from 2 L Polyethylene terephthalate (PET) bottles, into which bovine and pork offal 2-d- into decomposition were placed as baits. Traps were suspended 1.5 m above the ground.

Adult individuals were collected and transported to the laboratory. After removal from the traps, they were identified using the identification key of [38]. Subsequently, 10 adults were allocated in cages of 40 cm^2^ and held in an incubator at 27 ± 1°C and a 12:12 h light-dark cycle, and fed provisioned sugar and water *ad libitum*.

Bovine liver, bought in a city meat house, was used as a substrate for oviposition. After oviposition, the eggs were separated in lots of 0.05 g using a fine brush and transferred into a 300 mL glass container containing 200 g of ground beef. Fresh bovine liver was used as substrate for oviposition during the three experiments. After oviposition, the bovine liver was discarded, and 300 eggs were separated using a fine brush and transferred to a glass container of 300 mL capacity containing 200 g of raw ground beef. This amount of meat was enough for the development of all larval stages until the post-feeding phase. The container was covered with organdy cloth.

According to Wells [39] the pupation stage in this species starts on the fifth day, in this sense, after the larvae had fed, they were transferred on the fourth day, into a 500 mL glass bottle, in which 250 mL of dry sawdust was added as substrate for pupation.

The period from post-feeding until adult stage in this species lasts approximately five days in this temperature conditions [39]. Therefore, after adults have emerged 50 puparia from the first generation (F1) were separated for extraction of chemical compounds.

Similarly, in order to collect puparia from the F2 and F3 generations for chemical analyses, the same laboratory rearing and pupal exuvia collection procedure described for the F1 generation were used. The sources for the subsequent generations were from the emerged adults of the previous generation where 40 individuals (20 males and 20 females) from the same generation were collected and placed in the same cage, held at the same environmental conditions, and provisioned with sugar and water *ad libitum*. Raw bovine liver was offered for ovarian maturation of the specimens. After five days, bovine liver was added again for oviposition, with immature rearing using the aforementioned quantity of eggs and ground beef. Puparia were collected 1 day after adult emergence. Then, the compounds were extracted from the whole puparia. Similarly, in order to collect puparia from F2 and F3 generations for chemical analyses, the same procedure described for F1 was used.

In order to obtain the second generation, 40 adults (20 males and 20 females) were separated from the newly emerged of the first generation (F1) and kept in a cage of 40 cm^2^ for ovary maturation. They were fed with 10 g of raw bovine liver in a petri dish every day for five days. From the sixth to the tenth day, the adults were only fed water and sugar. On the eleventh day, flies began to be fed again with 10 g of fresh raw bovine liver in a petri dish as substrate for oviposition. After oviposition, eggs were separated with the aid of a brush, counted and placed in glass bottles, following the same methodology used for F1 generation. In order to obtain the third generation the same procedures described for the previous generations were performed from 40 adults (20 males and 20 females) of newly emerged of the second generation (F2).

### Analysis of samples by gas chromatography coupled to mass spectrometry (GC/MS)

From each generation of flies, 50 puparia were divided into 10 groups of 5 for each extraction of chemical compounds. The chemical compounds were extracted without the use of any fixative. Each sample was immersed in a glass container with 2 mL of hexane (Tedia, HPLC grade) for 2 min. After the withdrawal of the solute, samples were dried in a fume hood and stored in freezer at -20°C for a maximum of 30 days, for chromatographic analysis. For the analyses, each extract was solubilized in 200 μL of hexane.

Samples were analyzed on a gas chromatograph-mass spectrometer (GC-MS Ultra 2010, Shimadzu, Kyoto, Japan), with a DB-5 capillary column (J & W, Folsom, California, USA), (30 m length x 0.25 mm diameter x 0.25 μm film thickness). Analysis conditions were: injection volume of 1 μL in splitless mode; heating ramp with initial temperature of 150°C, ramped to 300°C at a rate of 3°C min^-1^ and a final hold at 300°C for 10 minutes. The injector temperature was set to 220°C. The temperatures of the detector and transfer line was 300°C. The mass spectrometer ion search was operated in electron ionization (EI) mode with -70 eV electrons, the mass analyzer was scanned from m/z 45 to 600, at 0.3 s per scan.

The identification of compounds was aided by using a calculated retention index [40], based on a standard of linear alkanes (C_14_-C_36_, Sigma Aldrich with purity ≥ 90%) and comparing the calculated value with the retention index in the literature [30,31,41–45], along with interpretation of mass spectra obtained from the samples and compared with mass spectra in the databases (NIST21 and WILEY229).

Compounds with less than 0.1% were not presented in the tables.

### Statistical analysis: Discriminant analysis

To assess whether there are significant differences between cuticular compounds in the puparia of three different generations, a discriminant analysis was applied using the relative areas of 100% of the compounds detected by chromatographic analysis.

The existence of significant differences between the generations was considered when p < 0.05; and Wilks’ Lambda was used as a measure of the difference between the groups, with values close to zero indicating no overlap between generations, while values close to 1 indicate high overlap and consequent lack of significant difference between them [46]. The analysis was carried out on Systat 11 software package.

## Results

**Fig 1:**
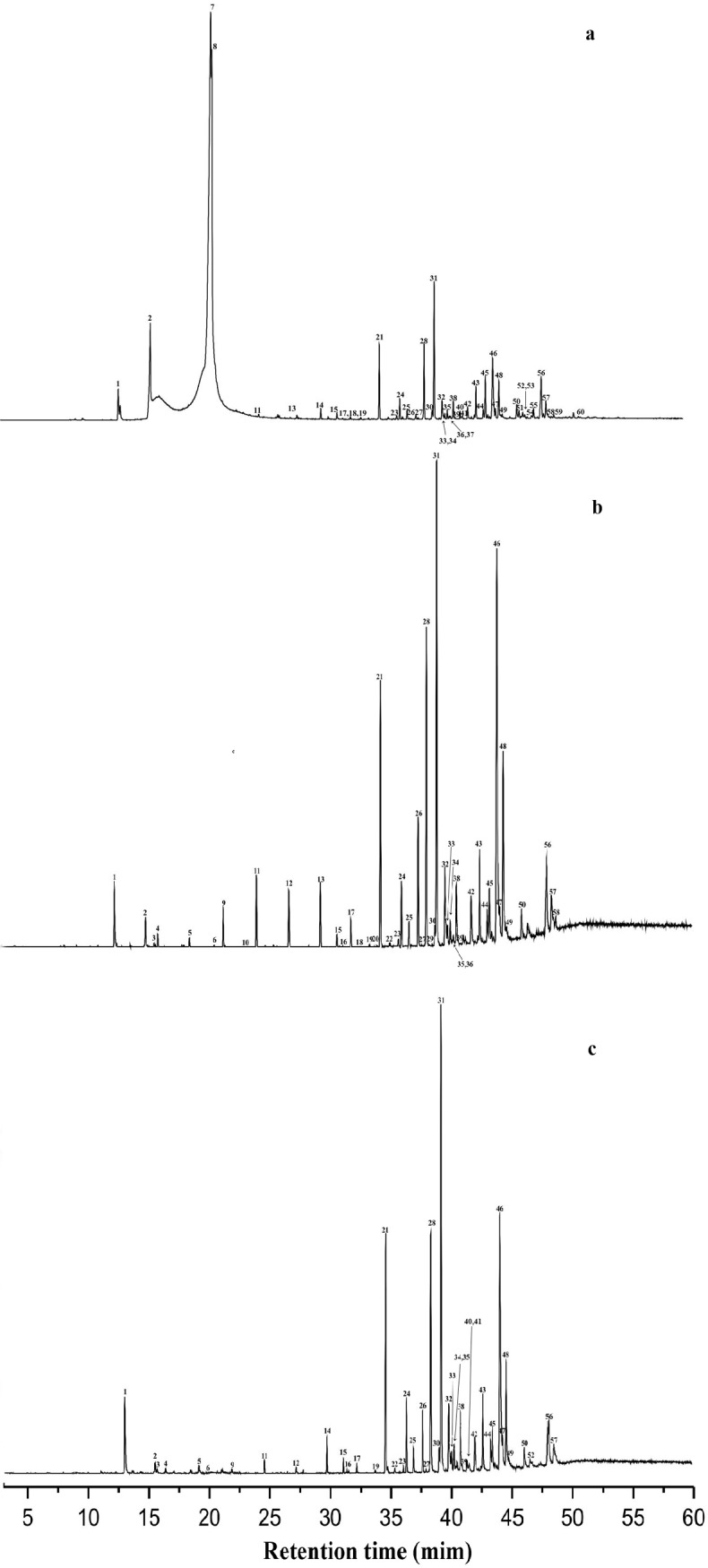
Representative profiles of compound present in empty puparia *Chrysomya megacephala*. (a) = GC chromatograms of 1st; (b) = GC chromatograms of 2nd and (c) = GC chromatograms of 3rd generation of the blow fly *Chrysomya megacephala*. Numbers refer to the substances listed in Table 1.

**Fig 2:**
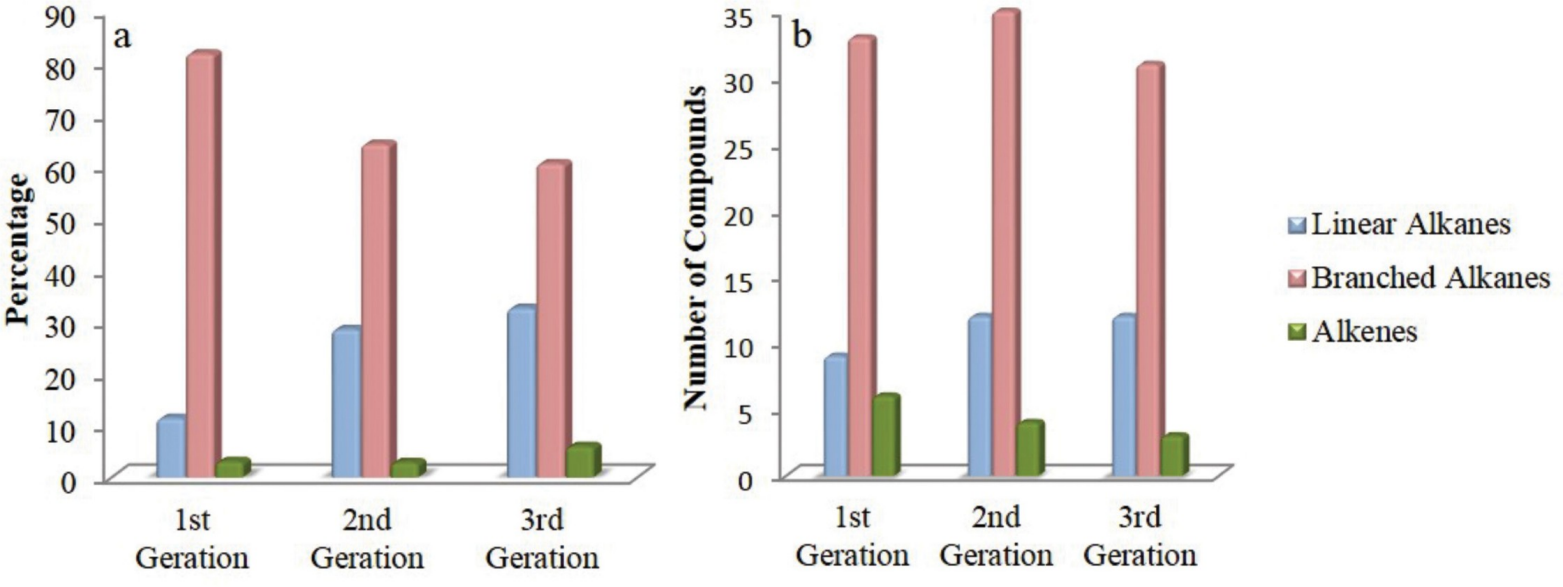
Bar graphic of compound classes present in empty puparia *Chrysomya megacephala*. (a) = Mean percentages of compound classes; (b) = Number of compounds present in empty puparia of three different generations of the blow fly *Chrysomya megacephala*.

A total of 85 peaks were detected in samples from the three generations; of these 60 were identified representing 96% of the relative area of all peaks detected. The compounds included a range from C_18_ to C_34_ (Table 1 and Fig 1). Of the identified compounds, 38 were common for all generations (Table 1 and Fig 2).

**Table 1:** Relative proportions of cuticular hydrocarbons present in empty puparia of three generations of the blow fly *Chrysomya megacephala*.

Peak	RT (min)	Compound	ECL	1st Generation (F1)	2nd Generation (F2)	3rd Generation (F3)
				Relative Abundance (% ± Standard Deviation)		
1	13.255	3- MeC_18_*	1875	1.90±0.27	2.04±1.45	2.42±0.75
2	15.769	2-MeC_19_*	1969	10.43±5.19	0.58±0.42	0.28±0.06
3	15.909	3-MeC_19_	1975	ND	0.42±0.77	0.19±0.07
4	16.546	C_20_	2000	ND	0.58±0.06	0.11±0.07
5	19.283	C_21_	2100	ND	0.28±0.30	0.21±0.04
6	20.180	9-MeC_21_	2131	ND	0.41±0.81	0.13±0.09
7	20.781	5-MeC_21_	2153	37.75±12.64	ND	ND
8	20.872	4-MeC_21_	2156	12.56±2.33	ND	ND
9	22.148	C_22_	2200	ND	0.52±10	0.18±0.12
10	24.163	C_23:1_	2278	ND	0.16±0.38	ND
11	24.769	C_23_*	2300	0.12±0.05	1.55±1.02	0.52±0.24
12	27.390	C_24_	2400	ND	1.19±0.71	0.24±0.31
13	29.512	C_25:1_*	2484	1.07±3.03	ND	ND
14	29.929	C_25_*	2500	0.40±0.11	1.26±0.66	1.24±0.34
15	31.270	7-MeC_25_*	2554	0.24±0.07	0.34±0.27	1.11±1.89
16	31.484	2-MeC_25_	2563	ND	0.95±1.86	0.15±0.12
17	32.398	C_26_*	2600	0.19±0.03	0.47±0.24	0.34±0.28
18	33.258	13-MeC_26_	2641	0.11±0.03	0.34±0.55	ND
19	33.897	3-MeC_26_*	2672	0.11±0.02	0.69±1.07	0.12±0.04
20	34.354	C_27:1_	2693	ND	0.96±1.56	ND
21	34.614	C_27_*	2700	3.01±1.31	7.41±1.20	8.61±1.47
22	35.555	7-MeC_27_	2741	ND	7.67±10.66	0.38±0.40
23	36.235	2-MeC_27_*	2767	0.43±0.82	0.81±1.16	0.21±0.07
24	36.490	C_28:1_*	2777	0.81±0.41	1.39±0.40	2.54±0.51
25	37.093	C_28_*	2800	0.41±0.13	0.74±0.25	0.76±0.18
26	37.828	14-MeC_28_*	2833	0.35±0.22	5.20±1.97	3.00±0.42
27	38.009	x-MeC_28_ *	2841	0.03±0.03	0.75±1.56	0.03±0.07
28	38.511	2-MeC_28_*	2863	2.60±1.14	8.30±1.46	9.59±1.06
29	38.966	4,12-DiMeC_28_	2884	ND	1.99±2.96	ND
30	39.183	C_29:1_*	2893	0.33±0.13	0.71±0.4	1.77±0.58
31	39.347	C_29_*	2900	5.48±2.11	12.56±4.81	16.97±3.21
32	40.001	13-MeC_29_*	2931	0.92±0.38	5.33±4.12	4.10±0.61
33	40.123	7-MeC_29_*	2937	0.11±0.03	0.26±0.24	0.04±0.13
34	40.233	9-MeC_29_*	2942	0.21±0.10	0.17±0.24	0.68±0.12
35	40.437	5-MeC_29_*	2951	0.33±0.16	0.50±0.12	0.86±0.15
36	40.597	11,15-;17,15-;9,17-;9,19-DiMeC_29_*	2958	0.12±0.03	0.53±0.86	0.13±0.09
37	40.703	11,19-DiMeC_29_*	2963	0.11±0.06	0.14±0.08	0.14±0.14
38	40.943	7,17-DiMeC_29_*	2974	0.70±0.32	1.11±0.51	2.84±0.71
39	41.077	5,13-DiMeC_29_*	2981	0.14±0.04	0.44±0.83	0.19±0.10
40	41.500	C_30_*	3000	0.26±0.09	0.23±0.11	0.40±0.10
41	41.685	3,9-;3,11-;3,13-DiMeC_30_*	3009	0.13±0.10	0.13±0.07	0.27±0.17
42	42.127	15-;14-MeC_30_*	3030	0.53±0.26	1.13±0.31	1.45±0.34
43	42.819	2-MeC_30_*	3063	1.39±0.78	1.98±0.71	2.68±0.42
44	43.427	C_31:1_*	3092	0.52±0.27	0.63±0.44	1.69±0.37
45	43.602	C_31_*	3100	1.41±0.39	1.77±0.94	3.05±1.70
46	44.205	15-;13-MeC_31_*	3130	4.93±2.57	12.46±5.27	17.34±1.53
47	44.432	7-MeC_31_*	3141	0.35±0.20	0.7±0.34	1.46±0.34
48	44.724	13,17-DiMeC_31_*	3155	1.59±0.73	4.3±1.68	4.88±0.78
49	44.999	9,17-;9,19-;9,21-DiMeC_31_*	3169	0.17±0.08	0.14±0.08	0.16±0.20
50	46.207	11-MeC_32_*	3229	0.68±0.37	0.69±0.31	0.86±0.15
51	46.399	10-;12-;13-;14-MeC_32_	3238	0.3±0.10	0.13±0.11	ND
52	46.701	12,16 -DiMeC_32_*	3254	0.24±0.17	0.12±0.16	0.23±0.18
53	46.872	2-MeC_32_	3262	0.25±0.22	ND	ND
54	47.437	C_33:1_	3291	0.2±0.17	ND	ND
55	47.605	C_33_	3200	0.13±0.08	ND	ND
56	48.237	13-MeC_33_*	3332	2.62±1.11	2.25±1.2	3.56±0.91
57	48.628	13, 17-DiMeC_33_*	3353	1.13±0.76	1.1±0.65	1.06±0.34
58	48.937	11,17,21-;11,17,23-TriMeC_33_	3370	0.004±0.01	0.19±0.16	ND
59	49.271	9,13,17-;9,15,19-;9,15,21;9,15,23-TriMeC_33_	3386	0.17±0.11	ND	ND
60	51.331	C_34:1_	3492	0.17±0.18	ND	ND

* = Compounds present in all generations; ND = Not detected; ECL = equivalent chain length; RT = Retention time.

From the first generation, 67 peaks were detected and 48 were identified that represented 96% of the relative area of all peaks detected, and of these 55 identified compounds, 8 compounds were only present in this generation. In generation 2, 67 peaks were detected, 51 compounds were identified representing 95.7% of the relative area of all peaks detected, and 3 of them were only found in this generation. From generation 3, 62 peaks were detected, 46 were identified representing 99.0% of the relative area of the peaks detected, and none of them was unique to this generation (Table 1).

**Fig 3:**
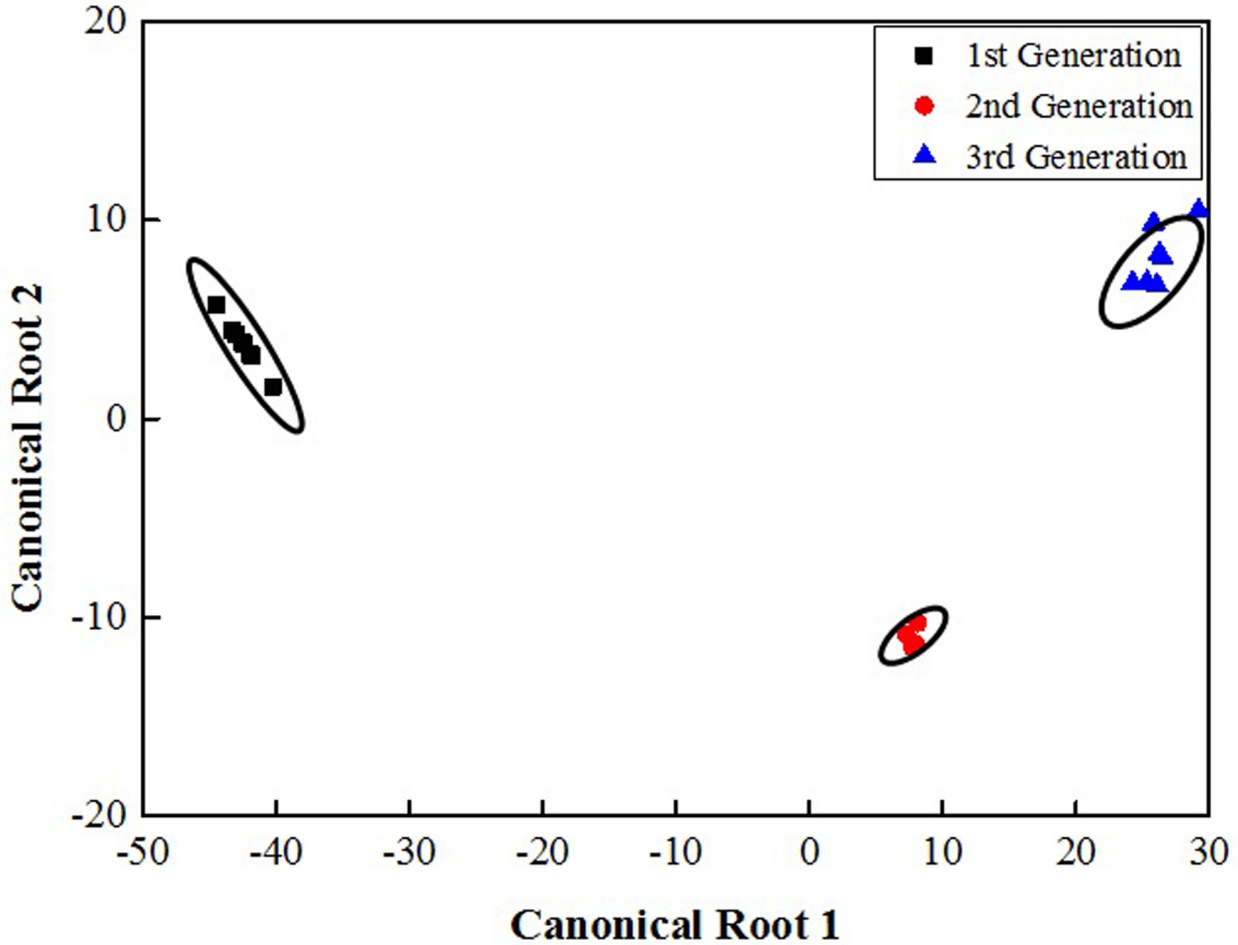
Dispersion diagram showing the results assessed by GC-MS of the specie *Chrysomya megacephala*. Differentiation of the cuticular chemical profile in empty puparia of three different generations of the blow fly *Chrysomya megacephala*.

There were both qualitative and quantitative differences between chemical compounds of puparia from the three generations (Table 1). The discriminant analysis (Fig 3) shows that, indeed, there are significant differences between compounds of different generations (Wilks’s Lambda = 0.001; F = 22.394 and p < 0.004).

The most prevalent compound classes, in order of importance in all samples, both according to the number and content were branched alkanes, followed by linear alkanes and then alkenes (Table 1 and Fig 2). Over the generations, it is possible to identify, in general, an increase in content and number of linear alkanes and a decrease in content of branched alkanes and number of alkenes (Fig 2).

## Discussion

According to the results, it is possible to distinguish F1, F2, and F3 generations of puparia by compounds present, as there were significant differences between them, and there are even exclusive compounds found between generation (Table 1 and Fig 1). Significant variation of chemical compounds in puparia from different generations has been described in *Hydrotaea aenescens* Wiedemann 1830 by [12].

In general, the identified compounds can be classified into three classes: linear alkanes, branched alkanes, and alkenes (Fig 2). Similar results were found by [30,31,47] in puparia of *C*. *megacephala* and *Chrysomya rufifacies* Macquart 1842. Ye et al. [10] evaluating the chemical composition of puparia of 6 species of flies also found similar classes of compounds. Of the three categories of compounds, there is a trend of increase in the percentage of the linear alkanes, probably because pupae need greater protection against desiccation [48].

Overall, the number of compounds of the puparia of the third generation was lower than the previous ones. A possibility for the decrease in number of compounds is inbreeding. According to Armold and Regnier [48] low genetic variability leads to reduction in number of cuticular compounds. This feature can cause homogeneity of cuticular profile of puparia along the generations, in addition to the decrease in exclusive compounds, as presented (Table 1). Thus, our results corroborate those of Menzel et al. [49], which identified a loss in cuticular chemical diversity caused by inbreeding in the ant *Hypoponera opacior* Forel 1893. Future studies that evaluate the influence of adding new individuals to the next generation are important to address this question.

Although in our study we did not analyze the effect of time on composition of puparia of a single oviposition cycle, according to Zhu et al. [30,31] in each successive generation, there is a tendency for the number of branched alkanes and alkenes to decrease, while there is an increase in linear alkanes over the generations. This pattern may be explained by the fact that pupae do not require compounds of more complex chains, those related to chemical signaling [50], such as branched alkanes. However, they require linear alkanes, which are fundamental to protect against desiccation [48].

Indeed, linear alkanes are major compounds in the pupal stage of flies. Armold and Regnier [48] reported that in the third larval stage, insects leave rotten flesh and search for a relatively dry environment, thus cuticular hydrocarbons provide protection against desiccation for pupae in the soil [48]. Gibbs [22] identified an increase in long chain cuticular hydrocarbons in *Drosophila melanogaster* Meigen 1830 samples subjected to desiccation relative to the control population, i.e. these flies change their profile based on the environmental conditions to which they are subjected.

Puparia of *C*. *megacephala* had substantially increased C_27_ and C_29_ with the progression of generations (Table 1 and Fig 1). Similarly, Goodrich [51] identified these two linear alkanes as the most abundant in puparia of the blow fly *Lucilia cuprina* Wiedemann 1830. Ye [10] also identified C_27_ and C_29_ in larger proportions in puparia of six species of flies of forensic importance. Gołębiowski [52] identified C_27_ and C_29_, besides C_31_ as the most abundant in pupae of *L*. *sericata*. In addition to linear alkanes, puparia of this species also contained the branched alkanes 2-MeC_28_ and 15; 13-MeC_31_ in relatively high contents in the three generations. These compounds are also found in considerable proportions in puparia of *Aldrichina grahami* Aldrich 1930 [10]. Indeed, these compounds are often found in samples of flies, especially in flies of forensic importance [53].

The importance of chemical compounds of fly puparia has been discussed by Ye et al.[10], which evaluated cuticular hydrocarbons present in puparia of six different species of flies of forensic importance and concluded that, since they vary significantly among species, both quali and quantitatively, with some compounds being more important for groups separation, therefore they can be suitable complimentary taxonomic tools. This result found by Ye et al. [10] is important because there are few morphological characters available, and the chemical components useful for analyses, such as DNA, decompose naturally over time.

Our results demonstrate that it is possible to distinguish puparia from different generations of blow flies by their chemical profiles (Fig 2). Considering that long chain cuticular hydrocarbons are relatively non-volatile [54] it is possible to use fragments of puparia and even stored samples as an alternative to speed up criminal investigations [12]. However, the climate, or local specific conditions should be considered, because colonization by more than one generation of flies might happen in places where the carcass takes a relatively longer time to decompose [32]. Thus, the preliminary studies performed in this study demonstrate that the analysis of puparia through chemical compounds appears to be a useful tool for one of the most important aspects of forensic investigations, the estimate of minPMI. This is the first step for validation and possibility of applying this method using puparia samples collected at crime scenes, where overlap of different generations is likely. However, future studies are needed using samples from more species under the effect of variable environmental conditions.
